# Gender‐Specific Molecular Pathways in Alzheimer's Disease: Insights from Proteomic Analysis

**DOI:** 10.1002/alz70855_103825

**Published:** 2025-12-24

**Authors:** Maria Luiza Hassdas Eiras, Vanessa V.J. De‐Paula

**Affiliations:** ^1^ Universidade Nove de Julho, Osasco, São Paulo, Brazil; ^2^ University of São Paulo (USP), Department of Psychiatry, Faculty of Medicine, IPq, HCFMUSP, Brazil

## Abstract

**Background:**

Alzheimer's Disease (AD) is a neurodegenerative disorder characterized by the accumulation of amyloid plaques, formed by the β‐amyloid peptide, and neurofibrillary tangles of Tau protein, leading to neuroinflammation and neuronal death. Clinically, AD begins with memory decline and progresses to deficits in judgment, orientation, and personality, ultimately resulting in dementia. The manifestation and progression of AD differ between sexes, with women representing two‐thirds of cases, exhibiting greater hippocampal atrophy and faster disease progression. At the molecular level, women have a higher burden of neurotoxic proteins and alterations in microRNAs, whereas men show more prominent inflammatory and apoptotic changes.

**Method:**

This study analyzed biological pathways and gender differences using brain tissue samples from the “Large‐scale proteomic analysis of Alzheimer's disease brain and cerebrospinal fluid” study. Of the 6,581 identified proteins, 2,344 were selected based on criteria such as sex, age, brain region, and the absence of Parkinson's disease or multi‐system atrophy. The proteins were grouped by gender and diagnosis (control, AD, and asymptomatic AD) and analyzed using the WebGestalt tool to identify biological pathways involved, focusing on those related to neuronal processes.

**Result:**

In **female controls**, pathways related to DNA repair and cellular senescence were prominent, such as “Presynaptic phase of homologous DNA pairing” and “Diseases of DNA Double‐Strand Break Repair.” In **male controls**, significant pathways involved ion transport, post‐translational modifications, and lipid metabolism, including “Ion transport” and “Metabolism of lipids.” In **female AD cases**, pathways related to membrane maintenance, cell cycle regulation, and post‐translational modifications were observed, such as “Anchoring fibril formation” and “Transcription of E2F.” In **male AD cases**, key pathways included “Thyroxine biosynthesis,” “ERBB4 signaling events,” and “ATF6 activation of chaperone genes,” related to protein maintenance and cellular stress responses. In **asymptomatic cases**, both genders shared pathways related to cell survival, metabolism, and differentiation. Females exhibited pathways like “ERBB2 activates PTK6” and “EGFR signaling,” while males showed pathways such as “Cell‐extracellular matrix interactions” and “MAPK signaling cascades.”

**Conclusion:**

The results indicate significant gender‐specific differences in the molecular pathways involved in Alzheimer's Disease, highlighting the need for further research to explore these differences and their implications for therapeutic development.

